# Effects of Short Forest Bathing Program on Autonomic Nervous System Activity and Mood States in Middle-Aged and Elderly Individuals

**DOI:** 10.3390/ijerph14080897

**Published:** 2017-08-09

**Authors:** Chia-Pin Yu, Chia-Min Lin, Ming-Jer Tsai, Yu-Chieh Tsai, Chun-Yu Chen

**Affiliations:** 1 School of Forestry and Resource Conservation, National Taiwan University, Taipei 10617, Taiwan; paulcmlin@gmail.com (C.-M.L.); tmj@ntu.edu.tw (M.-J.T.); 2 Graduate Institute of Oncology, College of Medicine, National Taiwan University, Taipei 10617, Taiwan; yctsai1@ntu.edu.tw; 3 Department of Tourism and Leisure Management, Yuanpei University of Medical Technology, Hsinchu 30015, Taiwan; samuel@mail.ypu.edu.tw

**Keywords:** forest bathing, pulse rate, blood pressure, heart rate variability, Profile of Mood States, State-Trait Anxiety Inventory, middle-aged and elderly

## Abstract

The present study investigated changes in autonomic nervous system activity and emotions after a short (2 h) forest bathing program in the Xitou Nature Education Area (XNEA), Taiwan. One hundred and twenty-eight (60.0 ± 7.44 years) middle-aged and elderly participants were recruited. Physiological responses, pulse rate, systolic and diastolic blood pressure, heart rate variability (HRV), and psychological indices were measured before and after the program. We observed that pulse rate, systolic and diastolic blood pressure were significantly lower after the program, which indicated physiological benefits from stress recovery. The Profile of Mood States negative mood subscale scores of “tension-anxiety”, “anger-hostility”, “fatigue-inertia”, “depression-dejection”, and “confusion-bewilderment” were significantly lower, whereas the positive mood subscale score of “vigor-activity” was higher. Furthermore, participants exhibited significantly lower anxiety levels according to the State-Trait Anxiety Inventory. However, changes in sympathetic and parasympathetic nerve activity were nonsignificant. Our study determined that the short forest bathing program is a promising therapeutic method for enhancing heart rate and blood pressure functions as well as an effective psychological relaxation strategy for middle-aged and elderly individuals.

## 1. Introduction

As human society becomes increasingly urbanized, various physiological and psychological diseases are caused by stress, thus affecting humans’ well-being and health [1,2]. Studies of leisure activities have identified the following benefits that potentially relieve stress and improve quality of life. Physiologically, leisure activities can relieve tension and fatigue, ameliorate disease, and enhance physical fitness to help people achieve health goals. Psychologically, leisure activities enable participants to relieve stress from life activities, relax mentally and physically, balance their moods, and obtain peak experiences. Socially, leisure activities improve the development of interpersonal relationships, harmonious relationships and friendships [3,4,5]. To improve health and well-being, studies have investigated various relaxation approaches such as yoga, massage, meditation, green attractions, and forest bathing [6,7,8,9,10,11,12,13,14]. In many Asian countries, forest bathing is a widespread outdoor activity and an alternative health-promotion approach [7]. Forest bathing or forest therapy refers to immersing oneself in nature and experiencing a forest’s atmosphere to improve mental and physical health [10,15,16,17,18,19]. A five-senses experience from walking or staying in a forest was reported to relieve stress and thus yield health benefits [20].

Several studies have determined that being in a forest environment has health benefits; participants in such an environment have lower pulse rates and diastolic and systolic blood pressures [6,7,21,22,23,24,25,26,27,28] than in urban settings. Decreased sympathetic nervous activity [7,20,22,23,24,29], cortisol levels [6,15,20,22,24,30], and salivary amylase activity [31] have been reported, as well as increasing parasympathetic nervous system activity [7,20,22,23,24,26,29]. Improved natural killer (NK) cell numbers and activity as well as percentages of granulysin, perforin and granzymes A/B have also been reported, indicating benefits to the human immune system [8,32,33,34]. The effect of forest visits on mental health has also been investigated in studies that have applied the Profile of Mood States (POMS) and determined that forest experiences increase positive emotions and reduce negative mood states [7,22,25,35,36,37,38,39,40,41] in comparison with urban stimuli. Some studies have also reported improvements in other psychological responses, including anxiety and depression [9,28,29,42,43,44].

Japan currently leads this field of research and has accumulated a large amount of scientific data on forests and human health. Substantial research on the Japanese practice of forest bathing (shinrin-yoku) has explored using forests for public health [45]. Taiwan aspires to diversify the use of forest services, particularly to promote forest and human health and well-being. For example, the Taiwan Forestry Bureau and the Experimental Forest of National Taiwan University (NTU) are developing forest bathing programs (e.g., Sensory Forest) for public use. Consequently, there is a need for understanding the health benefits of a specific forest bathing program. Additionally, considering diverse factors, such as forest type, environmental conditions, biodiversity, program content, and cultural background, this type of study is sensitive to the context of the environment, program, and participants, largely because of differences in trails, temperature and scenery. For example, the beneficial health effects derived from walking through a forest in Japan may differ from those derived from walking through a forest in Taiwan. Therefore, understanding the effects of forest bathing experience on human psychological and physiological responses in the local context is imperative. Furthermore, the major limitation of existing forest bathing research is small sample sizes and the fact that most studies have involved healthy (generally young and student) volunteers [10,44]. In order to make inferences at a population level, more research is required on the specific subgroups that might benefit most from forest exposure [45,46,47].

To this end, the present study investigated the autonomic nervous system activity and emotions of a large sample of middle-aged and elderly participants through field experiments and a one-group pretest–posttest design. Specifically, pulse rate, systolic and diastolic blood pressure, HRV, and mood states were measured before and after the popular short forest bathing program, Sensory Forest, which is provided by the Xitou Nature Education Area (XNEA). This study sought to understand the physiological and psychological effects of the short forest bathing program on middle-aged and elderly individuals.

## 2. Materials and Methods

### 2.1. Participants

One hundred and twenty-eight middle-aged and elderly subjects aged 45 to 86 years (60.0 ± 7.44; mean ± standard deviation) were recruited to the field experiment. The distributions of participants were 85 females (66.4%) and 43 males (33.6%); 59 subjects (46.1%) reported chronic diseases including diabetes, hypertension, heart and other diseases. The details and the frequency of natural recreation experience were reported in Table 1. All participants were capable of completing the forest bathing program and assessed initially to ensure that they had not consumed stimulants (i.e., alcohol and caffeine) prior to the experiment. The participants were fully informed about the study aims and procedures and voluntarily signed a participation agreement. A human subject’s compliance agreement was submitted to the Research Ethics Office of National Taiwan University; approval was received on 16 November 2015 (NTU-REC No. 201510HM015).

### 2.2. Study Sites

The field experiment site was in the planted forest mainly containing *Cryptomeria japonica* (1200–3000 per hectare) and *Phyllostachys pubescens* (3000–4500 per hectare) and the stand age ranged between 40 and 90 years old in XNEA, which is managed by the Experimental Forest of NTU and situated in central Taiwan. XNEA is a valley surrounded by mountains on three sides. The altitude of the forest area is from 800 to 2000 m; the temperature ranges from 11.0 to 20.8 °C (annual average temperature of 16.6 °C); and the humidity ranges from 88.0 to 93.0%. During the days of the forest bathing program, the weather was pleasant and it was not raining with a temperature of 22.6 ± 1.4 °C; 87.4 ± 3.8% relative humidity; wind speed of 0.1 ± 0.2 m/s.

### 2.3. Research Design

Participants were recruited from the XNEA main entrance. This study adopted a one-group pretest–posttest field experimental design to evaluate the autonomic nervous system activity and mood states incurred by the forest bathing program. Specifically, all participants were measured for their physiological and psychological responses before the forest bathing program (baseline), and post-program measures were collected upon their return. The effects of the forest bathing program on autonomic nervous system activity and mood states could therefore be identified by comparing the baseline and posttest measurements.

**Figure 1:**
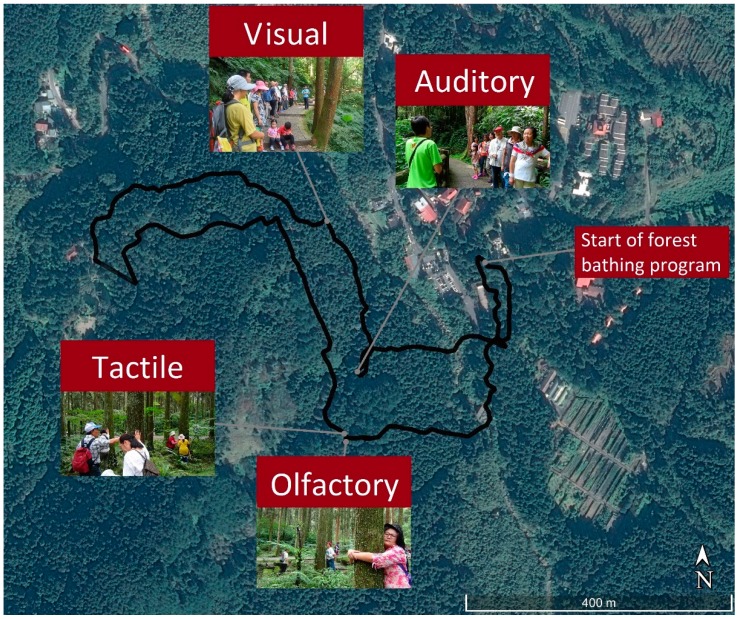
Walking course and various activities of the forest bathing program superimposed on a location map.

This study was conducted from 8:30 a.m. to 12:00 p.m. from 15 to 27 July 2016. Pretest and posttest data were collected from 8:30 to 9:15 a.m. and 11:15 a.m. to 12:00 p.m. The investigation was conducted once a day in the morning to limit changes in temperature, humidity, wind speed, and light levels. A guided 2 h forest bathing program (Sensory Forest) was organized to include the stimulation of four senses, namely visual (e.g., scenery), auditory (e.g., the sound of running streams or birds singing), olfactory (e.g., the smell of wood), and tactile (e.g., feeling the surfaces of leaves and trees) to facilitate the forest bathing experience (Figure 1). The total distance was 2.5 km, with an altitude of 1166–1192 m; the average walking speed was around 2 km/h and the walking course had a slight slope (5%). All participants completed the short trip smoothly. Consumption of alcohol, tobacco and caffeine was prohibited during the study period. The study set a maximum of 12 participants in each group that ensures quality of the program. The numbers of participants on each day were 8, 7, 6, 8, 8, 13, 16, 13, 10, 6, 11, 12 and 10 during 15–27 July 2016.

### 2.4. Physiological Indices

Pulse rate, systolic and diastolic blood pressure, and HRV were measured to assess autonomic nerve system activity. Pulse rate and systolic and diastolic blood pressure were obtained from the nondominant arm using a portable digital sphygmomanometer (EW-BW33, Panasonic Ltd., Osaka, Japan). The low-frequency (LF) and high-frequency (HF) data were measured using a handheld HRV monitor (LR-8Z11, Yang Ying Corporation Ltd., Taiwan). The HRV frequency spectrum comprises LF (0.04–0.15 Hz) and HF (0.15–0.40 Hz). The HF component is an estimate of parasympathetic nerve activity, whereas the LF/HF ratio reflects sympathetic nerve activity [30,48].

### 2.5. Psychological Indices

The Chinese versions of two psychological scales with satisfactory validity and reliability, namely the POMS and State-Trait Anxiety Inventory (STAI), were administered prior to and following the forest bathing program. The POMS was used to evaluate the psychological responses to forest therapy [49]. The POMS comprises 37 adjectives following six subscales: “tension-anxiety”, “anger-hostility”, “fatigue-inertia”, “depression-dejection”, “confusion-bewilderment”, and “vigor-activity.” Anxiety level was investigated using the STAI state anxiety subscale [50].

### 2.6. Data Analysis

All statistics analysis was performed using SPSS 20.0 (IBM Corporation, Armonk, NY, USA). A paired sample *t*-test was conducted to compare the differences between the pretest and posttest measurements. Data are expressed as the mean ± standard error (mean ± SE). In all comparisons, a *p*-value of <0.05 was considered statistically significant. Effect size was reported using Cohen’s d.

## 3. Results

**Figure 2:**
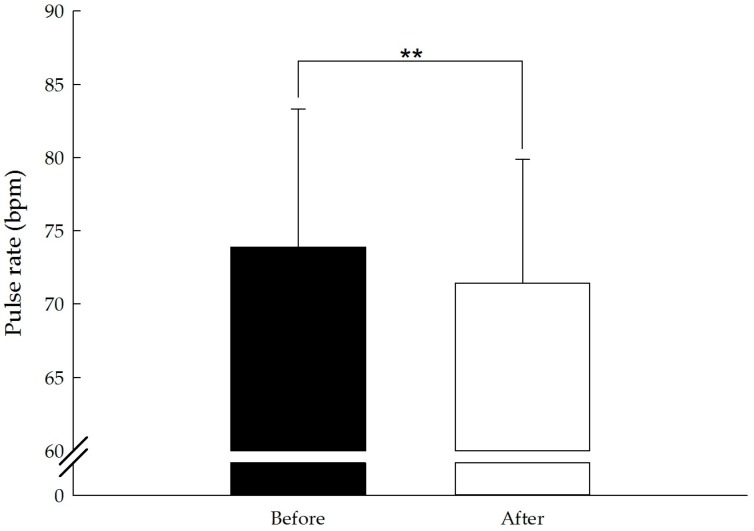
Effect of the short forest bathing program on pulse rate. N = 128, mean ± standard error. ** *p* < 0.01, paired-sample *t* test.

**Figure 3:**
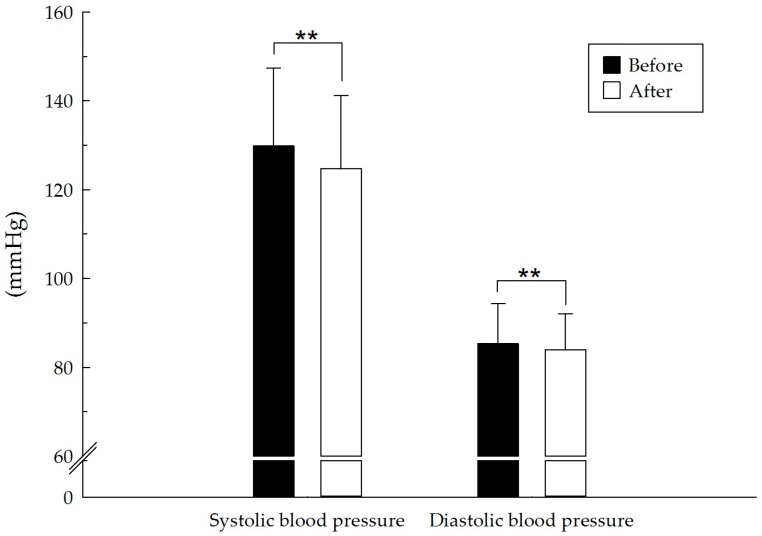
Effect of the short forest bathing program on systolic and diastolic blood pressure. N = 128, mean ± standard error. ** *p* < 0.01, paired-sample *t* test.

**Figure 4:**
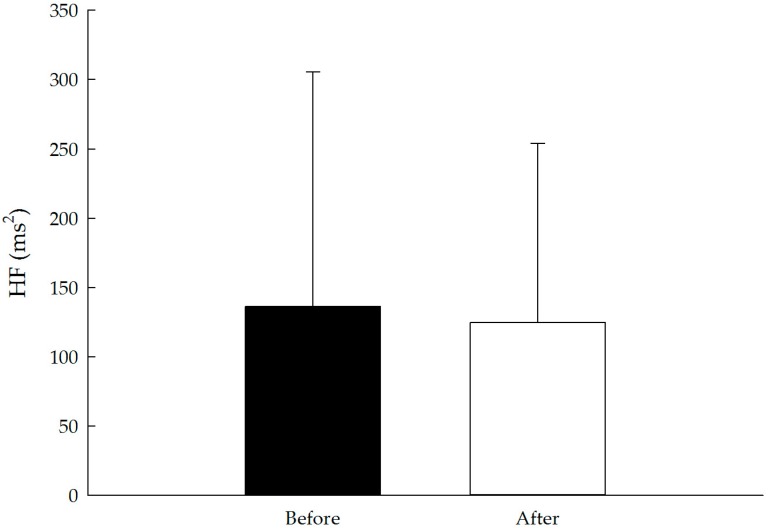
Effect of the short forest bathing program on HF. N = 128, mean ± standard error. Paired-sample *t* test.

**Figure 5:**
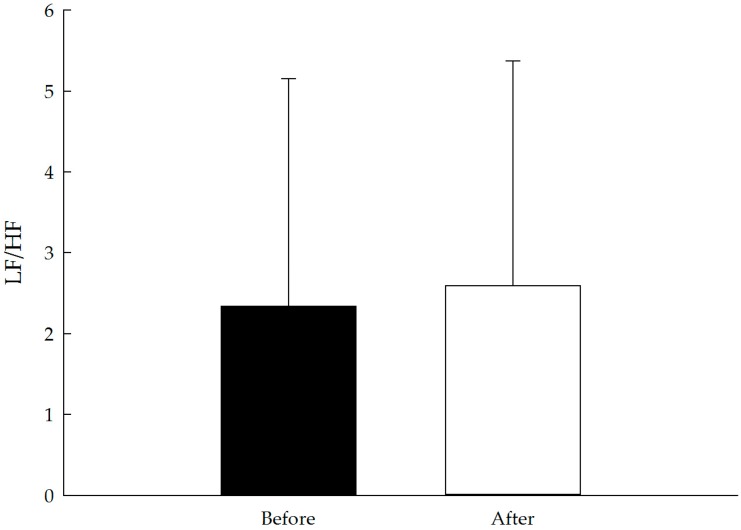
Effect of the short forest bathing program on LF/HF. N = 128, mean ± standard error. Paired-sample *t* test.

The results for pulse rate (pre 73.9 ± 9.4, post 71.4 ± 8.4 beats/min; t(127) = −4.82, *p* < 0.01, *d* = 0.43) and systolic (pre 129.9 ± 17.5, post 124.8 ± 16.5 mmHg; t(127) = −6.80, *p* < 0.01, *d* = 0.60) and diastolic blood pressure (pre 85.3 ± 9.1, post 84.4 ± 8.1 mmHg; t(127) = −2.70, *p* < 0.01, *d* = 0.24) were significantly lower after the forest bathing program. No significant change was observed in HF and LF/HF. The results of the physiological evaluation are presented in Table 2 and Figure 2, Figure 3, Figure 4 and Figure 5.

**Figure 6:**
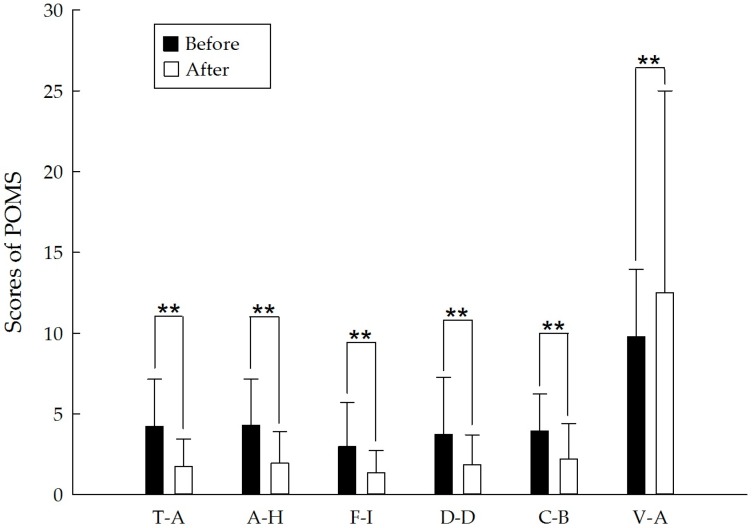
POMS scores before and after the short forest bathing program. T-A: tension-anxiety; A-H: anger-hostility; F-I: fatigue-inertia; D-D: depression-dejection; C-B: confusion-bewilderment; V-A: vigor-activity. N = 128, mean ± standard error. ** *p* < 0.01, paired-sample *t* test.

**Figure 7:**
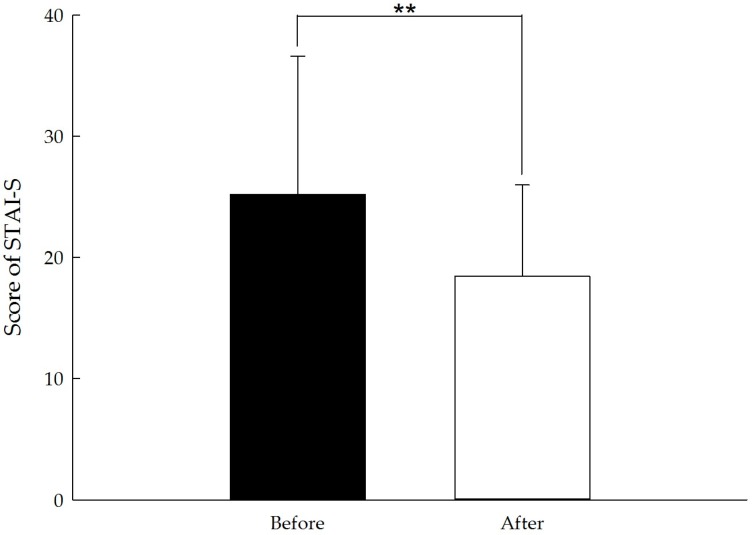
STAI-S score before and after the short forest bathing program. N = 128, mean ± standard error. ** *p* < 0.01, paired-sample *t* test.

All of the POMS and STAI scores revealed significant improvements after the forest bathing program. Five of the negative subscales of the POMS, “tension-anxiety,” “anger-hostility,” “fatigue-inertia,” “depression-dejection,” and “confusion-bewilderment,” decreased from pretest to posttest (all *p* < 0.01). Conversely, the positive mood state “vigor-activity” significantly increased (*p* < 0.01), and the state anxiety subscale in the STAI measurement exhibited improvements (*p* < 0.01). The POMS and STAI scores are presented in Table 3 and Figure 6 and Figure 7.

## 4. Discussion

This study explored the physiological and psychological responses to a short forest bathing program among middle-aged and elderly individuals. Pulse rate, systolic blood pressure, and diastolic blood pressure were determined to be significantly lower after the program. Previous studies have shown that heart rate and blood pressure are significantly lower in forest environments than in urban environments [6,7,19,23,24,25,26]. However, these studies have not indicated the efficacy of forest bathing per se in terms of stress reduction. Our findings indicated stress recovery as a result of the forest bathing program. Therefore, we concluded that the forest bathing program benefits pulse rate, systolic and diastolic blood pressure functions to middle-aged and elderly people.

Sympathetic and parasympathetic nerve activity can be determined by measuring HRV. In this context, most studies have reported that, compared with exposure to urban settings, exposure to forest environments results in higher parasympathetic and lower sympathetic nervous activity [7,15,22,29]. Our results offer a contrast at nonsignificant levels, namely a decrease in parasympathetic nerve activity and an increase in sympathetic nerve activity. It remains unclear as to why no significant change occurred in sympathetic and parasympathetic nerve activity in our study. It might be simply that the short forest bathing experience had limited effects on both the sympathetic and parasympathetic nervous systems. Or, unlike previous studies, sympathetic nerve activity and parasympathetic nerve activity were evaluated between time points within a single environment rather than between forest and urban environments in this study. Another possible explanation is that forest bathing experience combines enjoying the forest environment and walking in it. Enjoying the forest environment was expected to result in relaxation, which could increase parasympathetic nerve activity and suppress sympathetic nerve activity. However, physical exercise (i.e., walking or hiking) consumed individuals’ energy, and we suggested to participants that they pay attention to our program instructor; both of these factors may have boosted sympathetic nerve activity. We suggest that future studies explore potential variable-influenced physiological effects, for example, intensity of exercise (energy expenditure level), non-guided and guided forest bathing programs and/or environmental conditions (level of slope, humidity and atmospheric pressure). This information can greatly contribute to the development of the forest bathing program and forest management.

The POMS questionnaire results showed that the positive mood state of vigor was higher and the negative mood states of tension, anger, fatigue, depression and confusion were significantly lower after the forest bathing program. Additionally, state anxiety level decreased. These findings indicate that the short forest bathing program had psychological benefits for middle-aged and elderly people, which is consistent with the findings of previous studies [7,22,25,32,35,36,37,38,39,40,41]. Therefore, we concluded that the effects of psychological stress recovery of the forest bathing program extends to middle-aged and elderly people.

Forest bathing is an effective and convenient method of detaching oneself from urban environments. Undertaking activities in natural environments relieves stress and anxiety and thus improves health. Taiwan’s elderly population is growing rapidly. Currently, 13.3% of people are aged 65 years or older [51]. Evaluating the effects of the short forest bathing program on psychological and physiological benefits could help develop this type of program, provide proof of its effectiveness, and encourage the public to connect with nature. Furthermore, forest-based activities provide an opportunity for social interaction, allowing middle-aged and elderly people not only to enjoy the natural environment but also make new friends, which further contributes to their mental health. From a public health perspective, forest bathing is a simple and cost-effective method for improving health. Considering that 60% of Taiwan is forest-covered, forest bathing could be an effective health-promotion approach that could in turn reduce national medical expenses.

The present findings provide scientific evidence that the forest bathing program confers psychological and physiological benefits in middle-aged and elderly individuals by investigating a large sample size. Although some studies have suggested that longer forest bathing programs have a more beneficial effect [7], the present study clearly demonstrated that among middle-aged and elderly individuals, the short forest bathing program was associated with stress recovery. The short forest bathing program, Sensory Forest, could therefore become an effective forest therapy treatment and provide preventive benefits for stress-related illnesses to the specific group for enhancing their mental and physical health. However, the limitations of the present study include failing to collect information of confounding variables such as socio-economic status, medication usage, habits (e.g., smoking, exercise, etc.) and personality (e.g., nature lover), which potentially affect the physiological and psychological effects of forest bathing experience. Additionally, environmental factors such as forest aesthetics, types and levels of pollutions and environmental conditions should be included as covariates. Future studies should consider a range of subjects’ characteristics and/or micro-environmental conditions.

## 5. Conclusions

Our study revealed that the short forest bathing program elicited a significant decrease in pulse rate and systolic and diastolic blood pressure in middle-aged and elderly individuals. A short walk in the forest can reduce tension, anger, fatigue, depression, confusion and anxiety as well as improve positive emotion. However, sympathetic and parasympathetic nerve activity saw no improvement. In conclusion, the short forest bathing program contributes physiological and psychological healthy benefits on middle-aged and elderly people.

## Figures and Tables

**Table 1:** Summary of sample characteristics (N = 128).

Variable	Group	Number of Participants	Percentage
**Gender**	Male	43	33.6%
	Female	85	66.4%
**Chronic diseases**	Diabetes	9	7.0%
	Hypertension	25	19.5%
	Heart diseases	8	6.3%
	Other diseases	17	13.3%
	None	69	53.9%
**Natural recreation experience**	Once per month	40	31.2%
	2–3 times per month	37	28.9%
	More than 4 times per month	51	39.9%

**Table 2:** Effect of the forest bathing on physiological indices.

Physiological Indices	Pretest	Posttest	*p*	*t*	Effect Size
Pulse rate (bpm)	73.9 ± 9.4	71.4 ± 8.4	0.000 **	−4.82	0.43
Systolic blood pressure (mmHg)	129.9 ± 17.5	124.8 ± 16.5	0.000 **	−6.80	0.60
Diastolic blood pressure (mmHg)	85.3 ± 9.1	84.0 ± 8.1	0.008 **	−2.70	0.24
High-frequency (HF) (ms^2^)	136.5 ± 169.0	124.7 ± 129.3	0.345	−0.95	0.08
Low-frequency (LF)/HF	2.3 ± 2.8	2.6 ± 2.8	0.199	1.29	0.11

** *p* < 0.01, paired-sample *t* test. HF: high frequency; LF: low frequency.

**Table 3:** Effect of forest bathing on emotional state and anxiety.

Variables	Subscales	Pretest	Posttest	*p*	t	Effect Size
Emotional states (POMS)	tension-anxiety	4.22 ± 2.92	1.73 ± 2.19	0.000 **	−12.20	1.08
	anger-hostility	4.30 ± 2.87	1.95 ± 2.52	0.000 **	−10.95	0.97
	fatigue-inertia	2.98 ± 2.72	1.36 ± 1.95	0.000 **	−8.04	0.71
	depression-dejection	3.73 ± 3.52	1.85 ± 2.65	0.000 **	−7.70	0.68
	confusion-bewilderment	3.93 ± 2.30	2.20 ± 2.00	0.000 **	−10.46	0.92
	vigor-activity	9.77 ± 4.17	12.57 ± 4.47	0.000 **	9.00	0.80
Anxiety (STAI-S)	state anxiety	25.22 ± 11.39	18.44 ± 7.55	0.000 **	−8.45	0.75

** *p* < 0.01, paired-sample *t* test. POMS: the Profile of Mood states; STAI: State-Trait Anxiety Inventory.
